# Magnesium treatment in alcoholics: A randomized clinical trial

**DOI:** 10.1186/1747-597X-3-1

**Published:** 2008-01-25

**Authors:** Kari Poikolainen, Hannu Alho

**Affiliations:** 1 Finnish Foundation for Alcohol Studies, Helsinki, Finland; 2 National Public Health Institute (KTL), Department of Mental Health and Alcohol Research, Helsinki, Finland; 3 Research Unit of Substance Abuse Medicine, University of Helsinki, Finland

## Abstract

**Background:**

Magnesium (Mg) deficiency is common among alcoholics. Earlier research suggests that Mg treatment may help to normalize elevated enzyme activities and some other clinically relevant parameters among alcoholics but the evidence is weak.

**Methods:**

The effect of Mg was studied in a randomized, parallel group, double-blind trial. The patients were first treated for alcohol withdrawal symptoms and then received for 8 weeks either 500 mg of Mg divided into two tablets or matching placebo. Measurements were made at the beginning and in the end of the Mg treatment period. The primary outcome was serum gamma-glutamyltransferase (S-GGT) activity; secondary outcomes included aspartate-aminotransferase (S-AST) and alanine-aminotransferase (S-ALT) activity.

**Results:**

The number of randomized patients (completers) was 64 (27) in the treatment and 54 (31) in the control group. In intention-to-treat-analyses and in most analyses of study completers, there were no significant differences between the Mg-treated and placebo groups in the outcome variables. When baseline serum Mg level, coffee intake, and the number of unused Mg tablets were controlled for in a multivariate regression model, after-treatment serum Mg levels were found to be higher among the Mg-treated group than in the placebo group (t-test 3.334, df = 53, p = 0.002). After controlling for age, body weight, baseline alcohol intake, subsequent change in alcohol intake and baseline S-AST, the after-treatment S-AST levels were found to be lower among the Mg-treated group than in the placebo group (t-test 2.061, df = 49, p = 0.045).

**Conclusion:**

Mg treatment may speed up the S-AST decrease in compliant patients. This might decrease the risk of death from alcoholic liver disease.

**Trial Registration:**

ClinicalTrials.gov ID NCT00325299

## Background

Magnesium (Mg) deficiency is common among alcoholics [1-3]. Even in cases with normal serum Mg levels marked intracellular deficiency may be present. Animal studies have shown that Mg deficiency aggravates the hepatic damage caused by alcohol [4]. Mg treatment may help to normalize elevated enzyme activities and some other clinically relevant parameters, presented below, among alcoholics but the evidence is weak.

A Norwegian study on chronic alcoholics suggested that Mg treatment over six weeks decreases abnormally high activities of three enzymes related to liver function: serum gamma-glutamyltransferase (S-GGT), aspartate-aminotransferase (S-AST) and alanine-aminotransferase (S-ALT), and increases handgrip muscle strength [3]. The above results were, however, significant at the 5% level only when a 1-sided test was applied. This may be related to the small sample size, 24 alcoholics in the treatment and 25 in the control group. In the treatment for alcohol withdrawal syndrome Mg administration has been questioned [5], and a randomized study found no effect of intramuscular Mg injections on withdrawal symptoms [6]. However, long-term Mg treatment may help to restore liver function and other impairments after a drinking bout, but the evidence is not yet strong enough to warrant clear recommendations for clinical practice.

Mg deficiency may also be a cause of depression [7]. Mg ions regulate calcium ion flow in neuronal calcium channels, helping to regulate neuronal nitric oxide production. In Mg deficiency, neuronal requirements for Mg may not be met, causing neuronal damage which could manifest as depression. Rapid recovery from depression has been seen in some cases after Mg treatment [8]. Depressive symptoms are common among alcoholics during withdrawal from alcohol and often disappear during abstinence without any specific drug treatment [9,10]. It thus seems possible that Mg supplementation might also be useful in diminishing depressive symptoms among alcoholics after withdrawal. To clarify the clinical importance of the above, we studied the effect of oral Mg treatment in alcoholics, after treatment for alcohol withdrawal symptoms, in a randomized, parallel group, double-blind trial. Confounders were included in the analyses, when appropriate [11].

## Methods

### Planned study population and inclusion criteria

Eligible outpatients admitted to Töölö A-Clinic and Detoxification Unit (Töölön A-klinikka ja katkaisuhoitoasema in Finnish) in Helsinki, Finland, were invited to the study when they were about to complete their treatment for alcohol withdrawal symptoms. This unit treats out-patients with mild-to-moderate alcohol withdrawal symptoms. Diagnosis was based on drinking history and clinical examination. Rating scales and structured diagnostic interviews were not used. Day care activities included counseling, rest, recreational activities and medication, usually decreasing doses of benzodiazepines. Cases with delirium tremens or other alcohol-related psychosis were referred elsewhere for in-patient treatment. Thus the study group represents those alcohol withdrawal symptoms patients that can be treated in units providing conventional routine outpatient care. Research was carried out in compliance with the Helsinki Declaration. Ethical approval for the trial was granted by the Ethics Committee of the Helsinki and Uudenmaa Hospital District (Dno 80/E7/2002).

Based on earlier research the average decrease of S-GGT after Mg treatment was assumed to be 66 U/l and the population standard deviation (SD) 130 U/l (mean of Mg and placebo group SDs in [3]). At confidence level 95% (1-alfa) and power level 80% (1-beta), the sample size in both groups was set at 62 patients [12]. Allowing for 30% drop-out proportion the aim was to recruit two groups of 89 patients each.

Inclusion criteria were: admission to treatment because of an acute alcohol withdrawal symptoms or having an elevated S-GGT (men >80, women >50), no more Mg treatment within the past two months except ten 250 mg tablets or less, age 20–64 years, no history of heart rhythm disturbances, no contraindications against Mg treatment (e.g. heart failure, renal failure), normal serum creatinine and volunteering after informed consent. Moreover, patients had to have a fixed address and a telephone to facilitate follow-up. Follow-up appointment dates were agreed upon before the patients left treatment and they were contacted by telephone at least twice if they failed to keep the appointment.

### Intervention

The patients received orally for 8 weeks either a daily dose of 500 mg (20 mmol) of Mg tablets divided in two tablets (250 mg each) or matching placebo tablets. The tablets contained a mixture of magnesium carbonate, magnesium acetate and magnesium hydroxide. This mixture is the best compromise between a good uptake without any laxative effects. The magnesium acetate is valuable if someone has low stomach acid, because it can be absorbed even in this case. The tablets were dispensed in blister packs with 30 tablets in each.

### Outcome measures

The primary aim was to confirm that Mg treatment decreases elevated S-GGT among alcoholics. S-GGT was chosen to be the primary outcome of the trial because it is widely used in screening for alcohol problems and correlates with alcohol consumption. The secondary aims were to find out whether Mg treatment decreases the activity of S-AST and S-ALT, increases muscle strength and decreases depressive symptoms among these patients.

### Statistical analysis

Because both increase and decrease in the total body Mg are possible, 2-sided tests were considered to be more appropriate than 1-sided tests. Differences were considered significant at the level p < 0.05 (2-sided test). Differences for categorical variables were assessed with the Fisher's exact test. The independent sample t-test was applied for continuous variables between groups and the paired t-test for within-subject change. Main analyses focused on the end of Mg treatment values of the outcome variables. Both intention-to-treat and study completer (per protocol) analyses were made. Because the outcome variables were continuous, we report means, standard deviations and t-test significance values. Only the most relevant data findings are shown. Because some of the distributions seemed to deviate from normality, nonparametric Mann-Whitney test was also applied. However, the results were similar (data not shown). Because of the small sample size, randomization may not have guaranteed equal distribution of confounders between the study groups. Hence multivariate linear regression analysis was also applied to examine the relation of Mg treatment, adjusted for confounders, and enzyme activities at the end of the Mg treatment period of eight weeks. Because aminotransferases AST and ALT associate with alcohol intake and obesity, and weight increases by age, these factors were considered as confounders [11].

To see how the present results would add to the previous ones, a general variance-based fixed-effects model to a continuous measure of effect was applied by pooling the intervention effects with weights proportional to the inverse of the variance of the effect from the present and an earlier study [13].

### Assignment and blinding

Patients were randomized as individuals. Codes determining groups were derived from computer-generated random numbers and applied with the help of sealed opaque envelopes. Mg and placebo tablets were identical. Both groups were told of the possibility of mild diarrhoea as a side-effect. Code was kept secret by one of us (HA) and broken after the last patient had had his after-treatment examination.

### Measurements

Measurements were made at baseline and at the end of the 8 week treatment period. Alcohol intake was reported by answering standard questions on the habitual frequency and quantity of beer, cider, premixed drinks, wine and liquor during the past 30 days. When transforming these data into alcohol intake (g/day), alcohol contents were taken to be as follows: beer, cider and premixed drinks 3.6, wine 12 and liquor 30 g/l.

The self-administered 21-item Beck Depression Inventory (BDI) was used to assess the present depressive state of the respondent [14,15]. Each item included four or five graded statements and the respondents were asked to choose one or more options corresponding most closely with the actual condition at that time. Each item had numerical values from 1 to 3 indicating the severity of depressive mood and the total BDI score range was from 0 to 63. Scores 10–16 suggest mild depression, 17–29 moderate and 30 or more severe depression [14,15].

Maximal handgrip strength was measured in the sitting position with a Martin strain-gauge dynamometer and mean of three contractions was considered in data analysis. The reliability of this dynamometer has been found to be good [16]. Values less than 0.9 kp/cm^2 ^for men aged 50–54 years and less than 0.6 kp/cm^2 ^for women aged 45–54 years indicate less than average grip strength according to the reference values by the Institute for Occupational Health in Finland.

Blood pressure was measured in supine position with an automatic Omron M5-1 meter. A venous blood specimen was drawn for determination of S-GGT, S-AST, S-ALT, serum Mg and serum creatinine. Serum samples were transported in room temperature to the accredited laboratory (VITA Terveyspalvelut Oy, Helsinki, Finland) where the samples were analysed within a few days. Levels of S-GGT, S-AST, S-ALT and serum creatinine were measured by standard kinetic method based on the recommendation of European Committee for Clinical Laboratory Standards (ECCLS). Serum Mg was determined by a standard colorimetric method (Mg-Co, Boehringer Mannheim, Germany).

## Results

### Participant flow and follow-up

**Figure 1:**
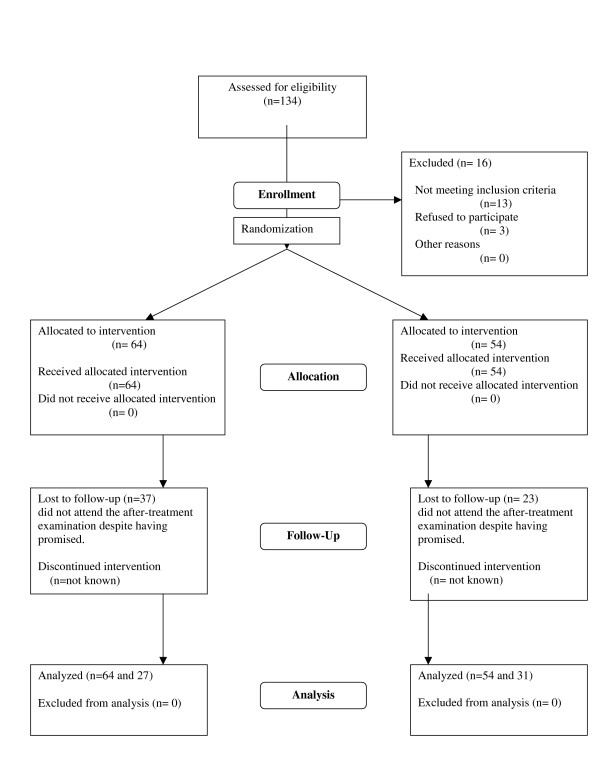
Participant flowchart.

Patient flow is shown in Figure 1. The final number of randomized patients was 64 in the treatment and 54 in the control group. Despite two or more telephone invitations to those patients who, in spite of their promise to attend, many subjects failed to keep their follow-up appointments. The follow-up examination was completed by 58 patients (49%). There were no differences between completers and dropouts in gender, education, self-rated health, systolic blood pressure, S-GGT, ALTor S-AST levels (Table 1). Completers were older, more often married and had higher diastolic blood pressure (p < 0.05).

**Table 1:** Baseline parameter values among completers and dropouts – Percentages or mean values (± standard deviation) and significance (*p*) of the difference

Parameter (Number of cases)	Completers (58)	Dropouts (60)	t-test	df	*p *value
Males, %	63.8	73.3			0.322^1^
Single, %	20.7	41.7			0.012^1^
Education < 10 years, %	25.9	30.0			0.684^1^
Average or poor self-rated health, %	65.5	61.7			0.705^1^
Age, years	48.7 ± 9.8	41.8 ± 10.3	3.714	116	<0.001
Alcohol intake, g/day	91 ± 93	141 ± 144	2.225	115	0.028
Beck Depression Inventory score	21.1 ± 8.0	20.5 ± 9.3	0.356	113	0.723
Systolic blood pressure, mmHg	139 ± 21.2	134 ± 17.1	1.456	116	0.148
Diastolic blood pressure, mmHg	93 ± 10.3	87 ± 12.3	2.896	116	0.005
Grip strength, right hand, kp/cm^2^	0.84 ± 0.25	0.87 ± 0.28	0.767	110	0.445
Grip strength, left hand, kp/cm^2^	0.82 ± 0.27	0.86 ± 0.31	0.782	115	0.436
Serum gamma-glutamyltransferase U/l	158 ± 371	120 ± 169	0.707	116	0.481
Serum aspartate-aminotransferase, U/l	62.9 ± 63	60.9 ± 41	0.205	116	0.838
Serum alanine-aminotransferase, U/l	55.0 ± 49	66.8 ± 59	1.176	116	0.242

^1^Fisher's exact test

### Analysis

In intention-to-treat-analyses, there were no significant differences between the Mg-treated and placebo groups in the outcome variables (data not shown). Of the completers, 27 patients were in the treatment group and 31 in the control group. Respectively, 502 or 16.6% and 749 or 21.6% of all trial tablets were returned. There were no significant differences between the treatment and control groups in change in S-GGT, ALT, S-AST, blood pressure, maximal handgrip strength or BDI (Table 2). Combining the present results and the earlier ones [3] by meta-analysis gave a non-significant difference of 3.7 U/l (mean, 95% confidence interval -30, 37) in S-GGT change compared with the placebo group.

**Table 2:** Mean change and significance (*p*) of the difference in study parameters eight weeks after treatment for alcohol withdrawal symptoms among 27 patients on magnesium 500 mg per day and 31 patients on matching placebo

Parameter	Magnesium	Placebo	t-test statistic	df	*p *value
Systolic blood pressure, mmHg	6.1 ± 18.3	2.1 ± 19.4	0.804	57	0.425
Diastolic blood pressure, mmHg	1.2 ± 9.8	-3.9 ± 10.6	1.922	57	0.058
Grip strength, right hand, kp/cm^2^	0.03 ± 0.09	0.0 ± 0.11	0.085	57	0.933
Grip strength, left hand, kp/cm^2^	0.03 ± 0.10	0.05 ± 1.27	1.319	57	0.192
Serum magnesium, mmol/l	0.06 ± 0.12	0.01 ± 0.08	1.801	56	0.077
Serum gammaglutamyl-transferase, U/l	-55.4 ± 105	-128.1 ± 49	0.752	56	0.455
Serum aspartateamino-transferase, U/l	-22.0 ± 43.8	-34.2 ± 79	0.714	56	0.478
Serum alanineamino-transferase, U/l	-12.0 ± 41.0	-24.4 ± 53	0.986	56	0.329
Beck Depression Inventory score	-9.1 ± 6.5	-12.2 ± 11	1.236	54	0.222

When baseline serum Mg level, coffee consumption and the number of unused Mg tablets were controlled for in a linear regression model, after-treatment serum Mg levels were found to be higher among the Mg-treated group than in the placebo group (t-test 3.334, df = 53, p = 0.002). After controlling for age, body weight, baseline alcohol intake, subsequent change in alcohol intake and baseline S-AST, the after-treatment S-AST levels were found to be lower among the Mg-treated group than in the placebo group (Table 3). The mean S-AST level (SD) was 30.7 (28.9) in the Mg treatment group and 37.6 (30.1) in the placebo group.

**Table 3:** The effect of magnesium treatment on serum aspartate-aminotransferase (S-AST), controlling for potential confounders: multiple linear regression analysis

Independent variable	Regression coefficient	Standard error	t-test statistic df = 49	p
Study group, Mg vs. placebo	-13.640	6.619	2.061	0.045
baseline alcohol intake	0.100	0.102	0.981	0.331
change in alcohol intake	0.120	0.106	1.130	0.264
baseline S-AST	0.052	0.051	1.020	0.313
age	-0.299	0.342	-0.874	0.386
weight, kg	-0.059	0.210	-0.281	0.780
Constant	40.495	22.116	1.831	0.073

## Discussion

We aimed to confirm earlier findings showing that Mg treatment decreases S-GGT and S-AST, and increases muscle strength in alcoholics. As to most outcomes, confirmation failed, although there were in the present study more patients and higher Mg dose (500 mg vs. 375 mg) than in the earlier study [3]. However, since alcohol intake level, its change, baseline enzyme levels, age and body weight may influence after-treatment enzyme activity, randomization may not guarantee balanced groups in a small study. After controlling for the possible confounders, the after-treatment S-AST levels were found to be lower in the Mg-treated group than in the respective placebo group among the study completers. AST has earlier found to predict all-cause and liver disease mortality in an eight-year follow-up study [11]. Thus, we found that Mg treatment may speed up the S-AST decrease in compliant patients. This might decrease the risk of death from alcoholic liver disease.

That not more than 16.6% of all Mg tablets were returned unused in the Mg treatment group suggests reasonably good compliance and that possible side-effects of Mg did not interfere with the treatment. After controlling for unused Mg tablets and other factors that might have had an effect on the Mg levels a significant association between Mg treatment and serum Mg levels was found.

Our study had several limitations. A major one was the small number of participants. Due to practical difficulties, low rate of volunteering and unexpected move of the treatment unit to a new location, the recruiting of patients had to be closed prematurely. Another problem was the large number of dropouts. Despite promises to the contrary, given during two or more phone calls, the dropouts did not attend the follow-up examination. Both these problems decreased the statistical power of the study to detect significant differences.

Assessment of depression was based on the BDI. This scale correlates closely with the more accurate Hamilton Rating Scale for Depression; coefficients have ranged from 0.61 to 0.87 across a variety of clinical populations [15]. However, since BDI is mainly used as a screening instrument and does not cover all aspects of depression, the present results should be verified by further research.

It is possible that some positive effects of the Mg may have been negated by high alcohol intake before the beginning of the study or by a relapse to alcohol consumption among some patients over the Mg treatment period. We aimed to take this possibility into account by controlling for the baseline alcohol intake, subsequent change in alcohol intake in multivariate analyses.

The lack of significant findings between Mg treatment and most outcomes suggest that either there is no effect or that the effect is so weak that a larger number of cases are needed to show the effect. As to S-GGT, the difference between the present and the earlier study results may be partly explained by the large inter-individual variation of the S-GGT response to ethanol that has been observed in a long follow-up of abstinent and not abstinent alcoholics [17].

## Conclusion

Mg treatment may speed up the S-AST decrease in compliant patients. This might decrease the risk of death from alcoholic liver disease.

## Competing interests

The author(s) declare that they have no competing interests.

## Authors' contributions

KP had the idea. KP and HA planned and designed the study, organized and supervised the data collection and finalized the manuscript together. KP analysed data and wrote the first draft.
