# Magnesium treatment in alcoholics: a randomized clinical trial

**DOI:** 10.1186/1747-597X-3-5

**Published:** 2008-02-27

**Authors:** Kari Poikolainen, Hannu Alho

**Affiliations:** 1 Finnish Foundation for Alcohol Studies, Helsinki, Finland; 2 National Public Health Institute (KTL), Department of Mental Health and Alcohol Research, Helsinki, Finland; 3 Research Unit of Substance Abuse Medicine, University of Helsinki, Finland

## Correction

After publication of our work [1], we noticed that the magnesium (Mg) content of the trial tablets was inadvertently given as 250 mg. The correct content was 200 mg. Thus the first sentence under the subtitle "Intervention" should read: The patients received orally for 8 weeks either a daily dose of 400 mg of Mg tablets divided in two tablets (200 mg each) or matching placebo tablets.

## Competing interests

The author(s) declare that they have no competing interests.

## Authors' contributions

KP had the idea. KP and HA planned and designed the study, organized and supervised the data collection and finalized the manuscript together. KP analysed data and wrote the first draft.
